# Simultaneous NOD1 and NOD2 Deletion in Hematopoietic Stem and Progenitor cells Promotes Long-Term Donor Chimerism

**DOI:** 10.1016/j.jtct.2026.01.031

**Published:** 2026-02-02

**Authors:** Alana A. Hawkes, Sashi G. Kasimsetty, Hojun Li, Gerald P. Morris, Sunil M. Kurian, Stephanie Almeida, Sean E. DeWolf, Dianne B. McKay

**Affiliations:** a Department of Immunology and Microbiology, Scripps Research Institute, La Jolla, California; b Department of Pathology, University of California San Diego, San Diego, California; c Department of Pediatrics, University of California San Diego, San Diego, California; d Department of Surgery, Division of Organ Transplantation, Scripps Clinic and Green Hospital, La Jolla, California; e Division of Pulmonary and Critical Care Medicine, University of California San Diego, San Diego, California; f Division of Hematology/Oncology, Rady Children’s Hosipital, San Diego, 92123, California

**Keywords:** NOD1, NOD2, HSPC reconstitution, Long-term chimerism, Stem cell transplantation, Oxidative phosphorylation/ROS

## Abstract

Hematopoietic stem cell transplantation (HSCT) remains limited by inefficient donor cell engraftment, which is influenced by inflammatory stress responses triggered during conditioning and immune reconstitution. While immune-mediated barriers have been extensively studied, the molecular regulators within donor hematopoietic stem and progenitor cells (HSPCs) that influence engraftment efficiency remain less defined.

NOD1 and NOD2 are cytosolic pattern recognition receptors (PRRs) traditionally known for sensing bacterial peptidoglycans and initiating innate immune signaling. However, emerging data suggest that NOD1/2 signaling may be directly activated by cellular stressors that intersect with cellular stress pathways critical to HSPC function. Our previous work has demonstrated that NOD1 and NOD2 play a critical role in modulating sterile inflammation, a key component of the transplant environment. This positions these PRRs as potential regulators of HSPC stress responses and survival during transplantation. Despite this, the cell-intrinsic role of NOD1 and NOD2 in donor HSPC engraftment has not been fully explored. Understanding how these innate immune sensors influence HSPC function in the absence of infection could reveal novel strategies to improve engraftment and long-term chimerism following HSCT.

This study aims to determine whether the cytosolic pattern recognition receptors NOD1 and NOD2 intrinsically regulate hematopoietic stem cell reconstitution and to define the underlying mechanisms by which their deletion may enhance donor cell persistence and chimerism in transplant settings. We evaluated NOD1/2 expression in murine HSPCs, tested reconstitution efficiency of wild-type (WT) versus NOD1/2 double knockout (NOD1×^−/−^) donor cells in syngeneic and allogeneic recipients, and performed competitive transplant assays to assess cell-intrinsic effects. In addition, we tested the effect of NOD1/2 deletion in HSPCs on long-term survival, allograft rejection and tumor immunosurveillance. Transcriptomic profiling was performed to define mechanisms underlying altered reconstitution.

HSPCs constitutively expressed NOD1 and NOD2, and their transcription was upregulated by endotoxin and NOD ligands. Genetic deletion of NOD1/2 significantly increased donor cell reconstitution and long-term chimerism in both syngeneic and fully allogeneic recipients, with NOD1×2′ cells outcompeting WT cells in competitive transplantation. Enhanced chimerism was observed across bone marrow, blood, spleen, and lymph nodes, without altering immune lineage differentiation. NOD1×2^−/−^ chimeras showed normal survival, intact alloresponsiveness, and preserved tumor immunosurveillance. Transcriptomic profiling revealed significant reprogramming of NOD1×2^−/−^ donor HSPCs, with highly significant alterations in mitochondrial metabolism, inflammatory signaling, and oxidative stress responses in the NOD1×2^−/−^ HSPCs, suggesting metabolic reprogramming as a mechanism of improved chimerism.

In conclusion, NOD1 and NOD2 act as innate immune checkpoints that restrict donor HSPC reconstitution. Their simultaneous absence enhances chimerism without compromising immune defense or tolerance and reprograms stem cell metabolism to favor survival. Targeting NOD1/2 signaling may represent a novel strategy to improve hematopoietic stem cell transplantation outcomes.

## INTRODUCTION

Engraftment after hematopoietic stem cell transplantation (HSCT) remains a major clinical challenge. Successful engraftment requires efficient homing of transplanted hematopoietic stem and progenitor cells (HSPCs) to supportive bone marrow niches, survival and expansion of HSPCs into hematopoietic lineages, and reconstitution of hematopoietic function. A fundamental goal for successful engraftment is that the transplanted HSPCs develop long-term effective hematopoiesis. Indeed, engraftment is the most important determinant of overall survival after stem cell transplantation [1,2].

A major barrier to successful stem cell engraftment is the cellular injury that donor hematopoietic stem cells sustain during their collection and transplantation. During procurement from peripheral blood or bone marrow, HSPCs are subjected to mechanical, oxidative, and inflammatory stressors that can activate innate immune sensors such as NOD1 and NOD2. Although traditionally recognized for detecting bacterial peptidoglycan motifs, NOD1 and NOD2 are increasingly understood to respond to diverse sterile stress signals that involve mitochondrial damage, metabolic perturbations, and endoplasmic reticulum stress [3]. These stress responses are further amplified by recipient conditioning regimens, which heighten inflammatory signaling and compromise stem cell viability. Because HSPCs express pattern recognition receptors (PRRs) that couple innate immune sensing to hematopoietic regulation, they are particularly vulnerable to these environmental insults. Upon transplantation, donor HSPCs encounter a broad array of danger-associated cues that engage PRRs, initiating molecular cascades that influence their survival, engraftment efficiency, and lineage differentiation.

Among the most studied PRRs expressed by hematopoietic stem cells are the membrane-bound Toll-like receptors (TLRs) and the cytosolic NOD-like receptors (NLRs), both of which exert critical control over HSPC fate and function. While TLR pathways in hematopoiesis have been extensively characterized, growing attention has shifted to NLRs as intracellular sentinels that detect microbial components as well as sterile stress signals arising from cellular damage or metabolic imbalance [4]. Notably, NOD2, which is expressed in freshly isolated human bone marrow–derived CD34^+^ cells [5], detects muramyl dipeptide (MDP), a breakdown product of bacterial peptidoglycan, while NOD1 senses dipeptide motifs derived from gram-negative bacterial cell walls [4]. Upon activation, both receptors recruit the adaptor kinase RIP2, initiating NF-*κ*B and MAPK signaling cascades that modulate cytokine production, metabolic programming, and stress resilience. Emerging evidence suggests that NOD1 and NOD2 contribute to hematopoietic homeostasis not only during infection but also under sterile inflammatory conditions, potentially influencing HSPC quiescence, proliferation, and regenerative potential [6,7].

Previous studies from our laboratory demonstrated that simultaneous deletion of NOD1 and NOD2 confers protection against ischemia-induced inflammation and tissue injury [8,9], and also mitigates graft-versus-host disease [10]. Importantly, dual deletion is required, as NOD1 and NOD2 exhibit functional redundancy because loss of one receptor can be compensated by the signaling activity of the other [10,11]. Our laboratory also showed that NOD1 and NOD2 play critical roles in mature T cell responses to alloantigen. The simultaneous absence of both receptors increased T cell death following alloantigen stimulation through stabilization of the tumor suppressor p53 and reduced expression of the E3 ubiquitin ligase MDM2, a negative regulator of p53. Functionally, these molecular alterations conferred significantly improved survival in a model of acute GVHD [10].

Building on these findings, we hypothesized that donor stem cells, which are subjected to inflammatory stressors during their procurement, would benefit from the absence of NOD1 and NOD2 signaling. Given their known crosstalk, we utilized bone marrow from NOD1/NOD2 double-deficient mice to test whether simultaneous deletion of these cytosolic receptors enhances engraftment efficiency and durable hematopoietic chimerism.

## METHODS AND MATERIALS

### Mice

All mice used in these experiments were housed in the vivarium at Scripps Research and approved for use by the Institutional Animal Care and Use Committee of the Scripps Research Animal Research Center. All animals were handled according to the recommendations of the Humanities and Sciences and the standards of the Association for Assessment and Accreditation of Laboratory Animal Care. BALB/cByJ and C57Bl/6J (B6) and C57Bl/6J CD45.1+ congenic strains were obtained from Jackson Laboratories, Bar Harbor MN. The NOD1×2-deficient mice (NOD1×2^−/−^) were obtained from J. Matheson at the Scripps Research Institute and confirmed to be on a B6 background by backcrossing >10 generations and by lack of rejection of skin transplants onto WT B6 mice. The NOD1×2 deficiency was also confirmed by the absence of the specific NODs by screening tail clips using primers specific for NOD1and NOD2 proteins, as previously published [9].

### Identification of NOD1 and NOD2 transcripts

RNA was isolated from wild-type (WT, C57Bl/6J) and NOD1×2^−/−^ hematopoietic stem and progenitor cells (HSPCs; LIN^−^c-KIT^+^SCA-1^+^) using the Quick-RNA MiniPrep Kit (Zymo Research, Irvine, CA). Complementary DNA (cDNA) was synthesized from 500 ng of total RNA using the Super-Script III Reverse Transcriptase Kit (Invitrogen, Carlsbad, CA) according to the manufacturer’s instructions. Quantitative reverse transcription PCR (RT-qPCR) was performed on a QuantStudio 3 (Applied Biosystems) or Eco Real-Time PCR System (Illumina, San Diego, CA) using SYBR Green detection (SsoAdvanced SYBR Green Supermix, Bio-Rad, Hercules, CA). Primer sets specific for NOD1 and NOD2 were obtained from GeneCopoeia (Rockville, MD). To verify amplification specificity, qPCR products were resolved on an agarose gel and visualized with GelRed (Biotium, Fremont, CA), which labels double-stranded DNA. Relative expression levels were calculated from threshold cycle (Ct) values normalized to housekeeping controls.

### Mixed Bone Marrow Chimeras

For allogeneic transplants, recipient Balb/cByJ mice (H-2K^d^) received sublethal irradiation with a single 3Gy dose using an X-ray source irradiator, followed 2 h later by i.p. injection with 1.5 mg of depleting anti-CD8*α* antibody (clone 53.67, Bio X Cell, West Lebanon, NH). The following day, mice received 2 mg of depleting anti-CD40L (clone MR-1) antibody (Bio X Cell) through i.p. injection. Approximately 2 h later, they received a retroorbital injection with Lin- BM cells isolated from donor wild-type (WT, B6) or NOD1×2^−/−^ mice (both H-2K^b^). For syngeneic transplants, C57Bl/6J (B6) mice were used as recipients and pre-conditioned by the same methods as above.

Donor bone marrow was harvested by flushing femurs and tibias with sterile PBS. The marrow was filtered through a 40 μm cell strainer, red blood cells were lysed, and lineage-positive cells were depleted by labeling with a cocktail of biotinylated antibodies [anti-CD3 (145–2C11), anti-GR-1 (RB6–8C5), anti-CD11b (M1/70), anti-CD19 (6D5), and anti-Ter119 (Ter-119); BioLegend, San Diego, CA), followed by incubation with streptavidin-conjugated magnetic particles (BD Biosciences, San Jose, CA). Magnetic separation was used to isolate the lineage-negative fraction, which was used as the stem cell-enriched population for transplantation (Supplementary Figure 1). The fraction was washed several times, resuspended in sterile PBS and injected IV into the primed hosts at a concentration of ~5E5 cells. The mice were returned to their cages and administered water with antibiotics (TMS-trimethoprim-sulfamethoxazole). Engraftment was confirmed at intervals after injection of the donor stem cells by staining peripheral blood with anti-H-2K^b^ (AF6–88.5) (to detect donor cells) and H-2K^d^ (SF1–1.1) (to detect host cells) (BioLegend, San Diego, CA) and analyzing by FACS. The presence of donor progenitor cells was assessed by harvesting bone marrow, thymus, LN and spleen at intervals after bone marrow transplant and FACS staining for the donor cells using anti-H-2K^b^ (AF6–88.5) vs H-2K^d^ (SF1–1.1). For syngeneic transplants, recipient WT C57Bl/6J CD45.1+ congenic mice were pre-conditioned as described above, and donor cells from WT C57Bl/6J CD45.2+ and NOD1×2^−/−^ CD45.2+ mice were harvested as described above. These stem cells were injected IV into the primed hosts at a concentration of ~5E5 cells. The presence of donor progenitor cells was assessed by harvesting peripheral blood at intervals after bone marrow transplant and FACS staining for donor cells using anti-CD45.1 (A20) and CD45.2 (104).

### Competitive bone marrow transplants

Host Balb/cByJ (H-2K^d^) mice were preconditioned by sublethal irradiation (3Gy) and injection with anti-CD8a (53–6.7) and anti-CD40L (MR-1) antibody, as described above in the methods for mixed bone marrow chimeras. Donor bone marrow was collected from the congenic C57Bl/6J CD45.1+ strain and from NOD1×2^−/−^ (CD45.2+ C57Bl/6J) mice and stem cells isolated using methods noted above. Equal cell counts of cells from NOD1×2^−/−^ and WT CD45.1+ (approximately ~5E5/strain) were injected intra-orbitally into the conditioned host mice. Engraftment was assessed at intervals after injection of the mixture of donor HSPCs by staining peripheral blood, bone marrow, lymph node and spleen for anti-H2K^b^ (AF6–88.5), anti-H2K^d^ (SF1–1.1), anti-CD45.1 (A20), anti-CD45.2 (104), anti-GR-1 (RB6–8C5), anti-CD19 (6D5), anti-CD135 (A2F10), anti-CD117 c-Kit (2B8), anti-Sca-1 (D7), and anti-CD150 (TC15–12F12.2) (all BioLegend, San Diego, CA).

### Stem cell stimulations and NOD1/2 expression

HSPCs were isolated from WT or NOD1×2^−/−^donor mice as described above. After isolation, the cells were washed several times and plated in 96 well round bottomed tissue culture plates and either treated with LPS (100ng/ml), muramyl dipeptide (MDP) (10ug/ml), L-Ala-*γ*-D-Glu-mDAP (Tri DAP) (10ug/ml), or anti-CD3+anti-CD28 (each 5ug/ml) for times indicated in the graphs or results, or not stimulated (all ligands from Invivogen, San Diego, CA). The cultures were placed in an incubator (37°C, 5% CO2) for 24 h. To detect alloantigen-induced proliferation, WT or NOD1×2^−/−^ HSPCs were isolated, as described above, and then cultured with irradiated (25Gy by X-ray source irradiator) and RBC lysed Balb/cByJ APCs (H-2K^d^), derived from spleens. Cell proliferation, induced by ligand or alloantigen, was measured by MTT reagent assay method (Promega Corp, Madison, WI). Relative expression of NOD1, NOD2 was detected by isolation of RNA from collected and washed cells after stimulation. RNA was purified using a Quick-RNA MiniPrep kit (Zymo Research Corp, Irvine, CA). The first strand of cDNA of each sample was synthesized from 500ng total RNA using a SuperScript III Reverse Transcriptase kit (Life Technologies, Carlsbad, CA) according to the manufacturer’s instructions. qPCR was run and quantified using an Eco Real-Time PCR System (Illumina, San Diego, Calif., USA) detecting the amplification of SYBR Green fluorescent signals using SsoAdvanced SYBR Green Supermix (Bio-Rad, Hercules, CA) and primer sets for NOD1 or NOD2 (GeneCopoeia, Rockville, MD).

### Graft versus tumor model

B16-F10 melanoma cells (H-2K^b^) were cultured to ~50% confluency (in logarithmic growth phase) and then trypsinized, washed and brought to a concentration of 1E6 cells/mL in sterile PBS. After 6-week generation, WT and NOD1×2^−/−^ mixed chimeras were anesthetized and hair removed by clippers on the lateral thoracic region. A 100ul cell suspension was injected subcutaneously and the mice were then returned to the cage and allowed to recover. The resulting tumor growth was measured daily using a caliper to measure tumor diameters. The experiment was terminated when the tumor grew to 10 to 12 mm or became ulcerated, at which point the animal was sacrificed.

### Allogeneic skin graft transplantation

Six weeks after HSPC transplant, recipient chimeric mice (WT or NOD1×2^−/−^ mixed chimeras) were anesthetized with ketamine (100 mg/kg)/xylazine (8 mg/kg) and the flank hair shaved off with electric clippers, to prepare a graft bed on the lateral thorax. The graft bed was prepared by removal of the epidermis and dermis to the level of the panniculus carnosus, keeping the vascular bed undisrupted. Equal sized pieces (3×5mm) of donor (WT or NOD1×2^−/−^) tail skin were placed into the vascular bed, leaving a margin of 1–2mm on all sides. Skin from host Balb/cByJ was placed in the same graft bed next to the WT or NOD1×2^−/−^ skin as a technical control. The grafted skin was then covered with sterile antibiotic (bacitracin) impregnated Vaseline gauze, covered with a bandage and then wrapped in cloth tape. The grafts were left undisturbed for 7 d. On d 7, the bandages and gauze were removed, and the grafts were photographed on a daily basis. Rejection was scored as 90% necrosis of the grafted tissue. Survival fractions were determined using the Kaplan-Meyer method. Comparison of survival curves was performed using the log rank test provided by the Prism software (Graphpad Software, La Jolla, CA). Median survival was also calculated using Prism software.

### Transcriptomic profiling of donor-derived HSPCs

To evaluate the transcriptional consequences of NOD1/2 deficiency following transplantation, donor-derived HSPCs were isolated from BALB/c (H-2K^d^) recipients 6 wk after transplant. Mice had received either wild-type (WT) or NOD1/2 double-knockout (NOD1×2^−/−^, H-2K^b^) donor bone marrow as described above. Lin- cells were harvested from tibias and fibulas of recipient mice, and the donor-derived cells were enriched using magnetic-activated cell separation (MACS) with antibodies specific for the H-2K^b^ antigen (Miltenyi Biotec). Total RNA was extracted from purified donor cells using the Quick-RNA MiniPrep kit (Zymo Research), and RNA quality was assessed by Bioanalyzer (Agilent). Bulk RNA sequencing libraries were prepared using the NEBNext Ultra II RNA Library Prep Kit (New England Biolabs) and sequenced on an Illumina platform to a depth of ≥30 million paired-end reads per sample in a single batch. Raw reads were aligned to the mouse reference genome (mm10) using the STAR aligner, and differential gene expression was determined with DESeq2. Pathway enrichment and network analyses were performed using the Advaita iPathway platform using a *p*-value from Over-Representation adjusted FDR. Differential expression was visualized with volcano plots (log_2_ fold change versus −log_10_ adjusted *p*-value), and gene set enrichment analysis (GSEA) was used to identify pathways significantly enriched in recipients of either WT or NOD1/2^−/−^ donor HSPCs.

### Statistical analysis

Data are presented as mean ± SD unless noted. Two-group comparisons used 2-tailed unpaired t-tests. Multi-group or time/tissue × genotype data used one- or 2-way ANOVA with Bonferroni post-hoc. Survival/skin-graft outcomes used Kaplan–Meier with log-rank tests. RNA-seq DE was by DESeq2 (Wald; FDR Benjamini–Hochberg); significance: adjusted *p* < .05 and | log2FC|≥1. Two-sided *α* = 0.05. Analyses: GraphPad Prism.

## RESULTS

### NOD1 and NOD2 are constitutively expressed in donor-derived hematopoietic cells, and their transcription is increased by endotoxin and NOD1/2 ligands

To determine whether the NOD1 and NOD2 receptors were constitutively expressed in the donor hematopoietic stem and progenitor cell (HSPC) population, BM cells from WT mice were isolated as LIN^−^cKIT^+^SCA1^+^ and the HSPC phenotype confirmed by FACS (Figure 1, Panel A). Donor cells from WT mice were analyzed for NOD1 and NOD2 transcripts by RT-qPCR, and amplicon specificity was confirmed by agarose gel electrophoresis. NOD1 and NOD2 expression in lymph nodes cells and NOD1×2^−/−^ HSPCs were used as positive and negative controls, respectively (Figure 1, Panel B).

Since preconditioning exposes HSPCs to conditions likely to influence transcription of the NOD1 and NOD2 receptors, we tested whether treatment with endotoxin (LPS) or with synthetic ligands for NOD1 [L-Ala-*γ*-D-Glu-mDAP (Tri DAP)] and NOD2 [muramyl dipeptide (MDP)] would increase transcription of NOD1 and NOD2 mRNA in the donor cells. As shown in Figure 1, Panel C, we found that exposure of WT cells to LPS increased transcription of both NOD1 and NOD2. Exposure to MDP+Tri DAP also increased NOD1 and to a lesser extent NOD2 expression in the WT cells. These data show that both NOD1 and NOD2 are expressed in the WT donor cells and that their expression is upregulated by LPS and NOD1 and NOD2 ligands.

### Simultaneous deletion of NOD 1 and NOD2 improves peripheral donor-derived hematopoietic reconstitution in syngeneic hosts

To evaluate the relative reconstitution efficiency of WT versus NOD1×2^−/−^ donor stem cells we first tested syngeneic hosts that were injected with ~5E5 of WT or NOD1×2^−/−^ donor bone-marrow derived cells (WT and NOD1×2^−/−^mice carried the CD45.2 + allele). The donor cells were injected retro orbitally into conditioned C57Bl/6J congenic (CD45.1+) recipient mice. The percent of CD45.2+ donor cells in peripheral blood of the recipients of the WT versus NOD1×2^−/−^ HSPCs was then compared at 6 wks after injection. As shown in Figure 2, significantly more NOD1×2^−/−^ donor cells were detected in the peripheral blood of syngeneic hosts, reflecting enhanced donor-derived hematopoietic reconstitution.

### Simultaneous deletion of NOD1 and NOD2 improves donor chimerism in allogeneic recipients

To evaluate whether simultaneous deletion of NOD1 and NOD2 enhances donor chimerism across a stringent allogeneic barrier, we compared chimerism following transplantation of donor cells from WT or NOD1×2^−/−^ (H-2K^b^) donors into allogeneic BALB/cByJ (H-2K^d^) recipients. Equal numbers of cells (~5E5/strain) from WT or NOD1×2^−/−^ donors were injected into recipients that had been preconditioned with sublethal irradiation (3 Gy) and CD8/antiMR-1 antibody depletion 1 d before injecting the WT or NOD1×2^−/−^ donor BM cells, a regimen previously validated to induce stable mixed chimerism in an allogeneic setting [12].

As shown in Figure 3, peripheral blood analysis revealed significantly higher levels of H-2K^b^^+^ donor cells at both 6 wk (Panel A) and 20 wk (Panel B) post-transplant in recipients of NOD1×2^−/−^ donor cells compared to WT. These data show that not only does the simultaneous absence of NOD1 and NOD2 significantly enhance chimerism, but the chimerism is sustained over time.

### Competitive stem cell transplants confirm improved NOD1×2 deficient donor-derived hematopoietic chimerism

To identify the relative engraftment of NOD1×2^−/−^ versus WT donor stem cells (both H-2K^b^) in the same host, we directly compared presence of the different donor cell groups in the bone marrow of recipient mice (BALB/c, H-2K^d^) using an in vivo competitive transplant assay. Equal numbers of cells (~5E5/strain) from NOD1×2^−/−^(B6, CD45.2+, H-2K^b^) and WT (B6, CD45.1+, H-2K^b^) donors were co-mixed and injected retro orbitally into conditioned BALB/c (H-2K^d^) recipient mice. Percent of H-2K^b^ donor cells from WT (CD45.2+) versus NOD1×2^−/−^ (CD45.1+) was com-pared in the bone marrow at 6 wk after injection by staining for the congenic markers. The results demonstrated that there were significantly greater numbers of NOD1×2^−/−^ than WT donor LSK (LIN^−^cKIT^+^SCA1^+^) (Figure 4, Panel B) and LT-HSPC (LIN^−^cKIT^+^SCA1^+^CD135^−^CD150^+^) (Figure 4, Panel C) cells detected in the recipient bone marrow.

These competitive transplantation data demonstrate that NOD1/2 deficiency confers a significant advantage, with NOD1×2^−/−^ donor stem cells outcompeting WT counterparts in establishing long-term hematopoiesis in allogeneic recipients.

### Chimerism in lymphoid tissue increased in the absence of NOD1/2

To assess lymphoid tissue–specific chimerism, we tracked donor-derived cells (H-2K^b^) within the lymphoid organs of allogeneic recipients (BALB/c, H-2K^d^) at serial time points following transplantation (Fig. 5). Shown in Panel A, significantly more NOD1×2^−/−^ than WT donor cells were detected in recipient bone marrow, suggesting improved engraftment and survival of the donor stem cells with the simultaneous absence of NOD1 and NOD2. Analysis of donor cells showed greater expression of NOD1×2^−/−^ HSPCs in the recipient peripheral blood (Panel B), peripheral lymph nodes (cervical, brachial, inguinal pooled) (Panel C) and spleen (Panel D). Donor cells were analyzed for phenotypic differences, but no significant variations were observed across T cell subsets (CD3, CD4, CD8), B cells (B220), macrophages (CD11c, F4/80), or regulatory T cells (FoxP3, CD25, CD45+) (Panel E).

Together, these data demonstrate that dual deletion of NOD1 and NOD2 increases the donor cells in multiple lymphoid compartments without altering immune lineage differentiation.

### Absence of NOD1 and NOD2 does not impair survival of chimeric mice and does not impair rejection or tolerance

We next examined whether NOD1×2^−/−^ HSPCs affected long-term survival or immune tolerance in chimeric recipients. As shown in Figure 6 Panel A, overall survival was comparable between mice reconstituted with WT versus NOD1×2^−/−^ HSPCs, indicating that loss of NOD1 and NOD2 did not confer additional lethality during the 300-d observation period.

Given that mixed hematopoietic chimerism can promote transplant tolerance in rodents and humans [12–18], we next tested for the acceptance of allogeneic skin grafts. BALB/c (H-2K^d^) donor skin was grafted onto chimeric recipients derived from either WT or NOD1×2^−/−^ HSPCs. As shown in Panel B, both groups rejected the grafts with similar kinetics, suggesting that the absence of NOD1 and NOD2 in the donor HSPCs did not alter allospecific tolerance to skin grafts.

To further probe immune tolerance, we evaluated rejection of syngeneic B16F10 melanoma (H-2K^b^) tumors injected into the dermis of chimeric mice. As shown in Panel C, tumor growth was comparable between WT and NOD1×2^−/−^ chimeras, indicating that deletion of NOD1 and NOD2 did not impair tumor immunosurveillance or promote tolerance to tumor antigens.

Together, these findings demonstrate that while NOD1×2^−/−^ stem cells enhance hematopoietic engraftment, their absence does not negatively impact survival or promote immune tolerance to allografts or tumors.

### NOD1/2 deficiency reprograms the transcriptional profile of donor-derived HSPCs

To evaluate the transcriptional changes associated with the NOD1/2 deficiency in recipients following transplantation, donor-derived HSPCs were isolated from BALB/c/c (H-2K^d^) recipients of either wild-type (WT) or NOD1/2 double-knock-out (NOD1×2^−/−^, H-2K^b^) 6 wk after the stem cell transplants. We selected 3 biological replicates per condition, which is powered to detect mean-ingful differences in these inbred mice under controlled environmental conditions that significantly reduced inter-animal variability. As shown in Figure 7, transcriptomic profiling revealed significant gene expression differences between WT and NOD1/2^−/−^ HSPC recipients. 1,582 differentially expressed (DE) genes were identified out of a total of 36,119 genes with measured expression. These were identified using a threshold of 0.05 for FDR corrected statistical significance (*p*-value) and a log fold change of expression with absolute value of at least 0.5.

The volcano plot (Panel A) highlights transcripts differentially expressed. The 1582 significantly differentially expressed (DE) genes are represented in terms of their measured expression change (x-axis) and the significance of the change (y-axis). The significance is represented in terms of the negative log (base 10) of the *p*-value, so that more significant genes are plotted higher on the y-axis. The dotted lines represent the thresholds used to select the DE genes: 0.5 for expression change and 0.05 for significance. The up-regulated genes (positive log fold change) are shown in red, while the down-regulated genes are blue. Principal component analysis (PCA) (Panel B, left) demonstrated clear separation between WT and NOD1/2-deficient samples, while sample-to-sample correlation heatmaps (Panel B, right) confirmed internal consistency within groups. Pathway enrichment analysis identifies MAPK signaling and oxidative stress–related responses as dominant signatures in recipients of NOD1×2−/− HSPCs (Panel C).

Over-representation analysis (ORA) of differentially expressed genes revealed significant enrichment of pathways involved in cellular stress responses and immune signaling. The top 5 pathways, ranked by adjusted *p*-value, included the MAPK signaling pathway, antigen processing and presentation, neurodegeneration-associated pathways, thermogenesis, and chemical carcinogenesis–reactive oxygen species. Among these, the MAPK signaling pathway showed the highest level of enrichment (*p* = 2.5 × 10^−7^; FDR = 7.7 × 10^−5^), consistent with transcriptional activation of stress-responsive and metabolic regulatory networks. The bubble plot depicts each pathway’s statistical significance (−log_10_ pORA) and impact score (−log_10_ pAcc), with bubble size proportional to the number of genes represented within each pathway. These results highlight the MAPK cascade and redox-related processes as central nodes linking innate immune signaling and metabolic adaptation in NOD1/2-deficient stem cells. Pathway bar plots (Panels F and G) further showed enrichment of oxidative stress–related and mitochondrial pathways in NOD1/2-deficient HSPCs, supporting a role for NOD1/2 signaling in regulating metabolic reprogramming after transplantation.

Together, these data indicate that NOD1/2-deficient HSPCs undergo marked transcriptional reprogramming, characterized by altered mitochondrial stress responses and metabolic reprograming.

## DISCUSSION

Our findings identify NOD1 and NOD2 as key innate immune sensors that function as intrinsic barriers to efficient hematopoietic stem and progenitor cell (HSPC) reconstitution. We show that NOD1 and NOD2 are constitutively expressed in donor-derived HSPCs and that their transcription is further induced following exposure to endotoxin or synthetic NOD ligands. These data establish that HSPCs are not passive targets of inflammation but instead actively engage innate immune sensing pathways. Importantly, they suggest that transplant-associated inflammatory cues directly activate NOD1/2 signaling within donor HSPCs, thereby impairing stem cell function and reconstitution efficiency.

This model is biologically plausible in the transplant setting. Myeloablative conditioning is well known to induce sterile inflammation and activate innate immune receptors [19]. Although synthetic ligands were used experimentally, NOD1 and NOD2 can be triggered by a broad range of endogenous stress signals, including those associated with endoplasmic reticulum stress [20,21]), supporting the relevance of these pathways in the transplant setting.

Functionally, we found that simultaneous deletion of NOD1 and NOD2 significantly enhanced donor cell reconstitution in both syngeneic and allogeneic recipients. Increased chimerism was evident early after transplantation and persisted long-term, indicating that loss of NOD1/2 signaling supports durable hematopoietic reconstitution. Competitive transplantation assays further demonstrated that NOD1/2-deficient donor HSPCs outcompeted WT cells within the same host establishing a clear cell-intrinsic advantage. This advantage extended across multiple lymphoid tissues without altering lineage differentiation, suggesting that the NOD1/2 deficiency primarily affects engraftment efficiency and stem cell survival rather than fate determination.

The observation that mature hematopoietic lineages contribute equally from WT and NOD1×2−/− donors despite marked differences in total bone marrow and peripheral blood chimerism likely reflects the distinction between stem cell engraftment and downstream differentiation. We propose that NOD1×2−/− HSPCs engraft more efficiently within bone marrow niches, generating a larger overall pool of donor-derived cells. Once engraftment and differentiation occur, however, lineage allocation proceeds normally, resulting in comparable proportions of mature T cells, B cells, myeloid cells, and Tregs. Thus, equivalent lineage distribution does not imply equivalent stem cell contribution. Rather, NOD1/2 deficiency increases the quantity of engrafted stem cells without altering differentiation programs. This interpretation is supported by transcriptomic analyses showing that NOD1/2-deficient HSPCs undergo reprogramming of stress-response, MAPK, and oxidative metabolism pathways—changes consistent with enhanced stem cell fitness and niche persistence.

Chronic inflammation may further modulate these effects. Sustained exposure to cytokines, DAMPs, and microbial products can metabolically and epigenetically reprogram HSCs [22], while altering the threshold and kinetics of NOD1/2 signaling [23]. Understanding how chronic inflammatory priming reshapes NOD1/2 responses—and whether this results in tolerance, exhaustion, or competitive advantage of specific HSC subsets—represents an important direction for future investigation.

A major translational concern when enhancing engraftment is whether manipulation of innate immune pathways compromises immune surveillance or tolerance. Notably, simultaneous deletion of NOD1 and NOD2 did not impair long-term survival, allospecific rejection, or tumor immunosurveillance. Chimeric recipients of NOD1/2-deficient donor cells rejected allogeneic skin grafts and syngeneic tumors with kinetics comparable to WT controls, indicating that enhanced engraftment does not come at the expense of immune competence. These findings suggest that NOD1/2 signaling represents a therapeutic window in which engraftment can be improved while preserving essential immune functions.

From a translational perspective, these results are highly relevant to solid organ transplantation. Mixed hematopoietic chimerism has emerged as a powerful strategy to induce long-term tolerance and enable withdrawal of immunosuppressive therapy [24]. Mixed chimerism approaches pioneered by Strober, Sykes, Sachs and others [12] have enabled tolerance induction in preclinical models and facilitated immunosuppression withdrawal in clinical kidney transplantation [13,14,17,25]. By enhancing durable donor chimerism without impairing immunity, disruption of NOD1/2 signaling in donor HSPCs may represent a novel approach to improve the safety and efficacy of such protocols.

Although equal numbers of WT and NOD1×2−/− HSPCs were transplanted, WT cells are likely more vulnerable to injury from endogenous and exogenous danger signals that activate pattern recognition receptors during conditioning. This interpretation is consistent with our prior work in ischemic kidney injury, where combined deletion of NOD1 and NOD2 protected tissues from PRR-mediated damage [9], and aligns with the established observation that preconditioning regimens generate proinflammatory signals capable of activating multiple PRRs [26].

The role of NOD1 and NOD2 in transplantation is context dependent [10,27]. Prior studies have shown that NOD2 can function as a costimulatory receptor in CD8^+^ T cells [28,29], while promoting apoptosis in regulatory T cells [30,31]. More broadly, NOD receptors have been implicated in the regulation of cell death pathways. Structurally related to the apoptosome adaptor Apaf-1, NOD1 and NOD2 can activate caspase-9–dependent apoptosis [32]. NOD2 promotes Fas-mediated apoptosis in T cells and regulates Treg survival [30], while NOD1 engages caspase and JNK signaling pathways [33]. At the same time, both receptors can activate NF-*κ*B–dependent anti-apoptotic programs, underscoring their bimodal effects depending on context and signaling thresholds [34]. Together, these findings support a model in which NOD1/2 signaling exacerbates HSPC stress and apoptosis during transplantation, whereas their absence enhances donor cell persistence and engraftment.

Mechanistically, transcriptomic profiling revealed that NOD1/2 deficiency induces broad reprogramming of donor HSPCs, with enrichment of pathways related to oxidative phosphorylation and reactive oxygen species metabolism. These data suggest that innate immune sensing directly influences HSPC metabolic state and that NOD1/2 signaling imposes a stress program that compromises engraftment potential. By relieving this constraint, NOD1/2-deficient HSPCs may maintain a metabolic profile that favors survival and repopulation under transplant-associated stress.

In summary, our findings establish NOD1 and NOD2 as innate immune checkpoints regulating donor HSPC engraftment. Dual deletion enhances engraftment and chimerism without impairing immune defense and is associated with metabolic reprogramming that supports stem cell survival. These data highlight the translational potential of targeting NOD1/2 pathways to improve outcomes in hematopoietic stem cell transplantation, particularly in allogeneic settings.

Future studies will be required to define the precise signaling intermediates linking NOD1/2 activation to mitochondrial dysfunction and to evaluate whether pharmacologic inhibition of NOD1/2 can recapitulate the engraftment benefits observed with genetic deletion.

## STUDY LIMITATIONS AND FUTURE DIRECTIONS

Several limitations warrant consideration. First, the endogenous inflammatory cues that drive NOD1/2 induction in HSPCs following conditioning remain incompletely defined. Ongoing studies are profiling cytokine and DAMP signatures within the bone marrow immediately after conditioning and exposing purified HSPCs to physiologically relevant mediators to identify signals that most robustly induce NOD1/2 expression.

Second, the conditioning regimen used here includes anti-CD40L–based immunomodulation, which may create a specific inflammatory context influencing NOD1/2 signaling. Future studies will assess the generalizability of our findings across alternative conditioning strategies, including total body irradiation–based regimens.

Finally, while increased peripheral blood chimerism provides strong evidence of enhanced donor-derived hematopoietic reconstitution, it does not directly quantify bone marrow–resident HSPC engraftment. Direct enumeration and functional assessment of donor-derived HSPCs within the bone marrow niche will be an important focus of future work.

## Supplementary Material

1

Supplementary material associated with this article can be found in the online version at doi:10.1016/j.jtct.2026.01.031.

## Figures and Tables

**Figure 1.**
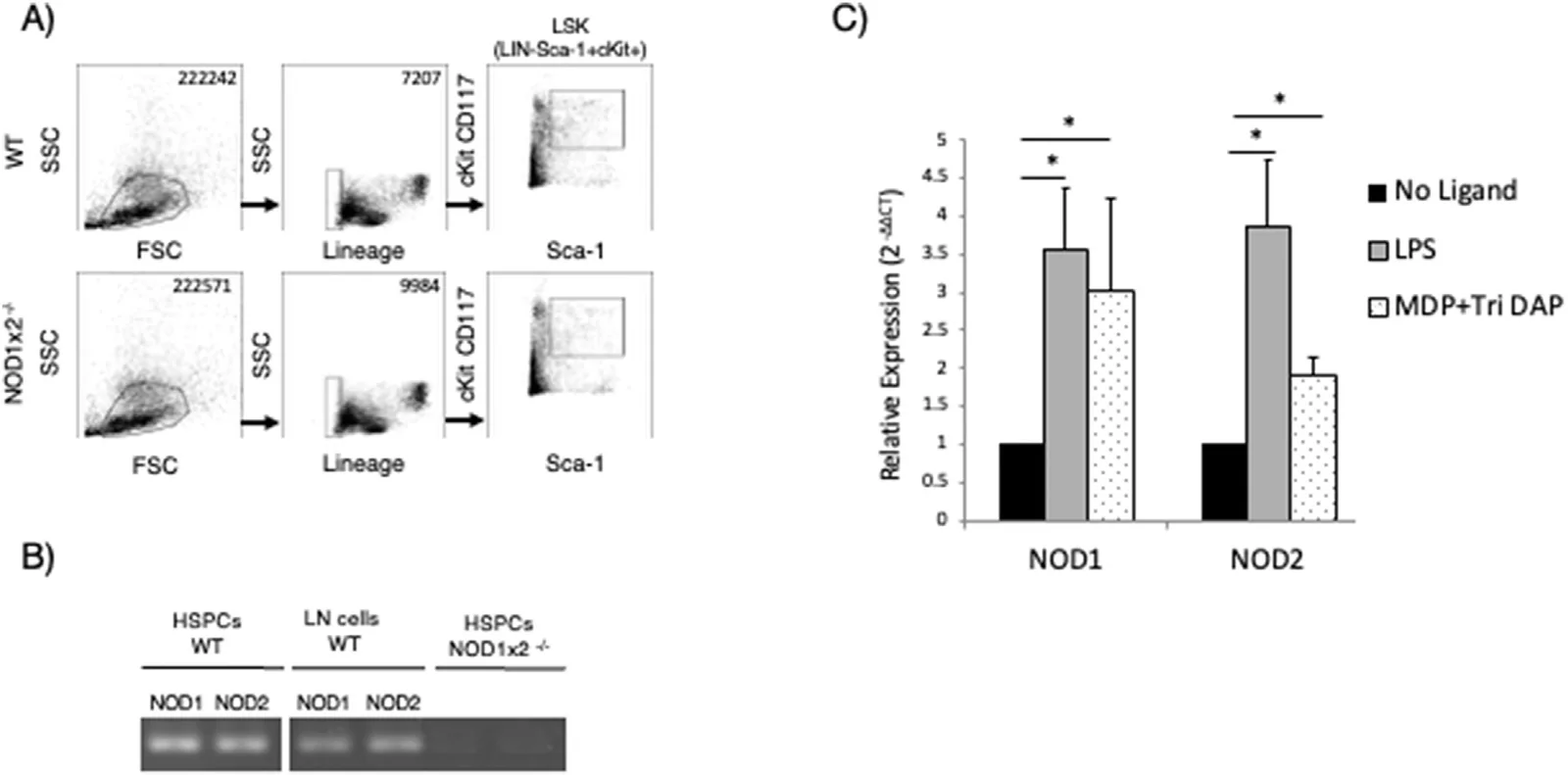
Donor stem cells (HSPCs) from WT mice express NOD1 and NOD2. (Panel A) Gating strategy used to isolate LIN- SCA-1+cKIT+ HSPCs from bone marrow of WT versus NOD1×2^−/−^ mice. (Panel B) Expression of NOD1 and NOD2 mRNA in WT HSPCs and LN cells and in NOD1×2^−/−^ HSPCs. The HSPCs negatively selected from bone marrow depleted of progenitor cells, and lymphocytes were isolated from peripheral LNs of WT mice. Total RNA was isolated from corresponding cells and specificity confirmed on agarose gel. (Panel C) Relative expression of NOD1, NOD2 mRNA in HSPCs stimulated for 24 h with ultrapure LPS (100 ng/mL) (grey bar) versus the NOD1/2 ligands MDP+Tri-DAP (each 10 ug/mL) (stippled bar) detected by qRT-PCR. Each graph represents 1 of 3 identical experiments. Error bars represent SD, *P < .05.

**Figure 2.**
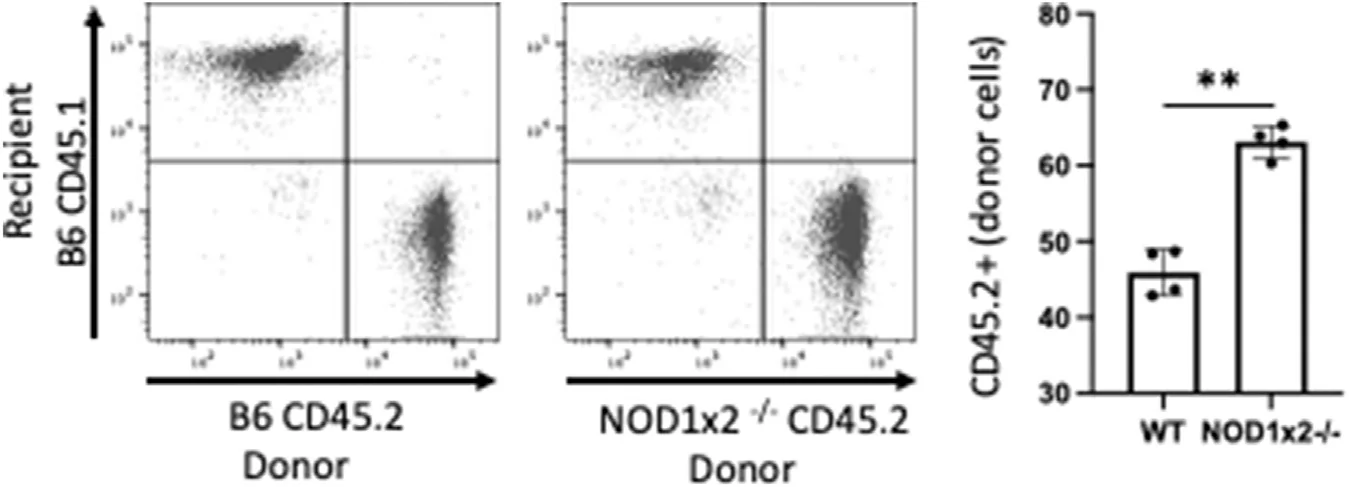
WT versus NOD1×2^−/−^ donor HSPCs transplanted in syngeneic hosts. (Panel A) Representative dot plots of donor (CD45.2+) cells in periphery after being injected 6 wk earlier into B6 (CD45.1+) recipient mice. (Panel B) Percentage WT versus NOD1×2^−/−^ donor (CD45.2+) cells in syngeneic recipients (CD45.1+) at 6wk. Error bars are SD and represent n=4 mice/gp, data is representative of 2 identical experiments, ** *p* <.01.

**Figure 3.**
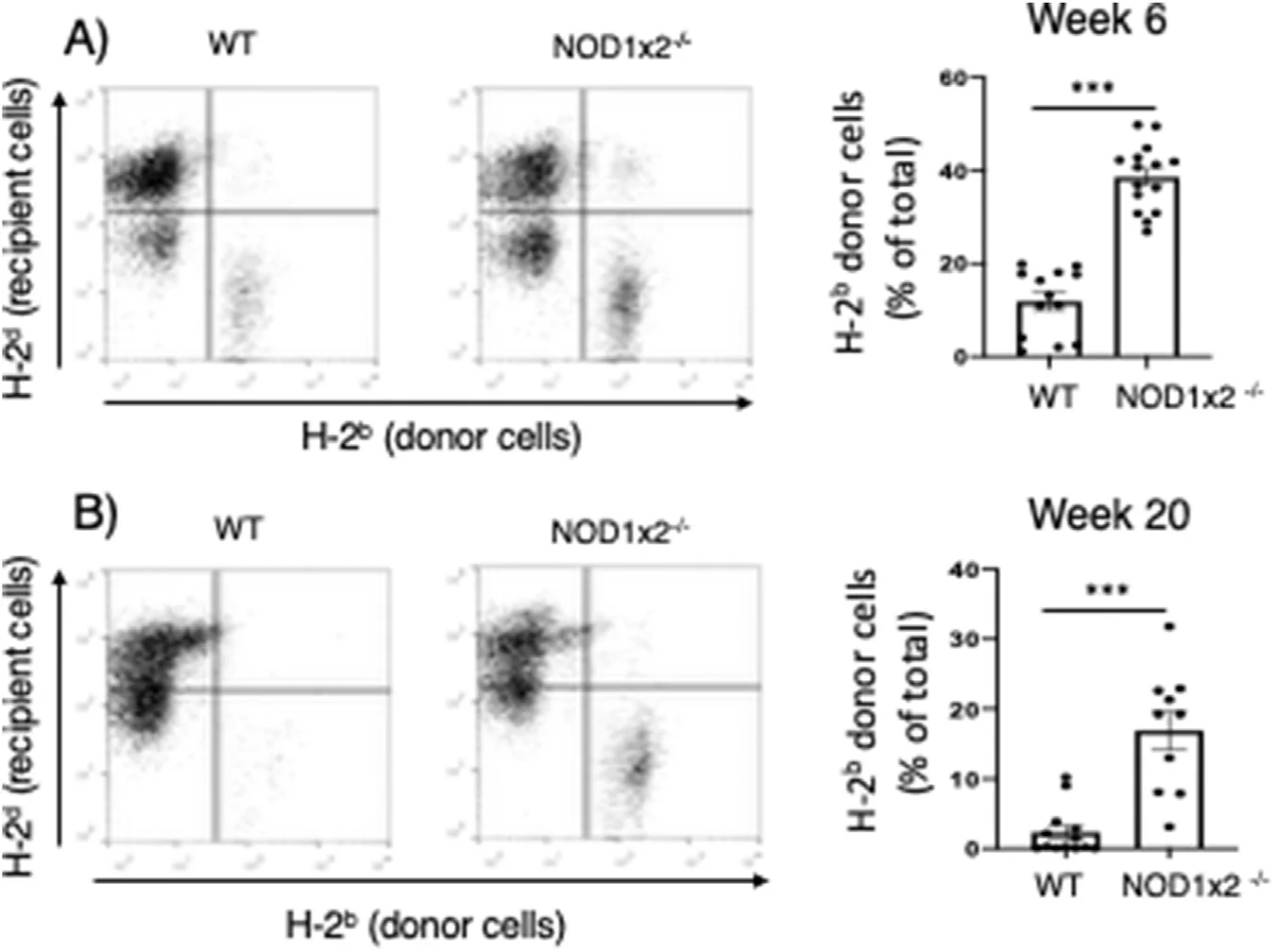
WT versus NOD1×2^−/−^ donor HSPCs transplanted into allogeneic hosts. (Panel A) Donor cells (H-2K^b^) in peripheral blood 6 wk after allogeneic hematopoietic stem cell transplantation into conditionally treated and irradiated BALB/c (H-2K^d^) mice (n = 13 WT [black bar], n = 15 NOD1×2^−/−^ [grey bar] chimeric mice/gp). (Panel B). Donor cells in peripheral blood 20 wk after allogeneic hematopoietic stem cell transplantation into conditionally treated and irradiated BALB/c (H-2K^d^) mice (n=13 WT (black bar), n=10 NOD1×2^−/−^ (grey bar) chimeric mice/gp). Error bars are SD, each experiment was repeated 4 times, ***P < .001.

**Figure 4.**
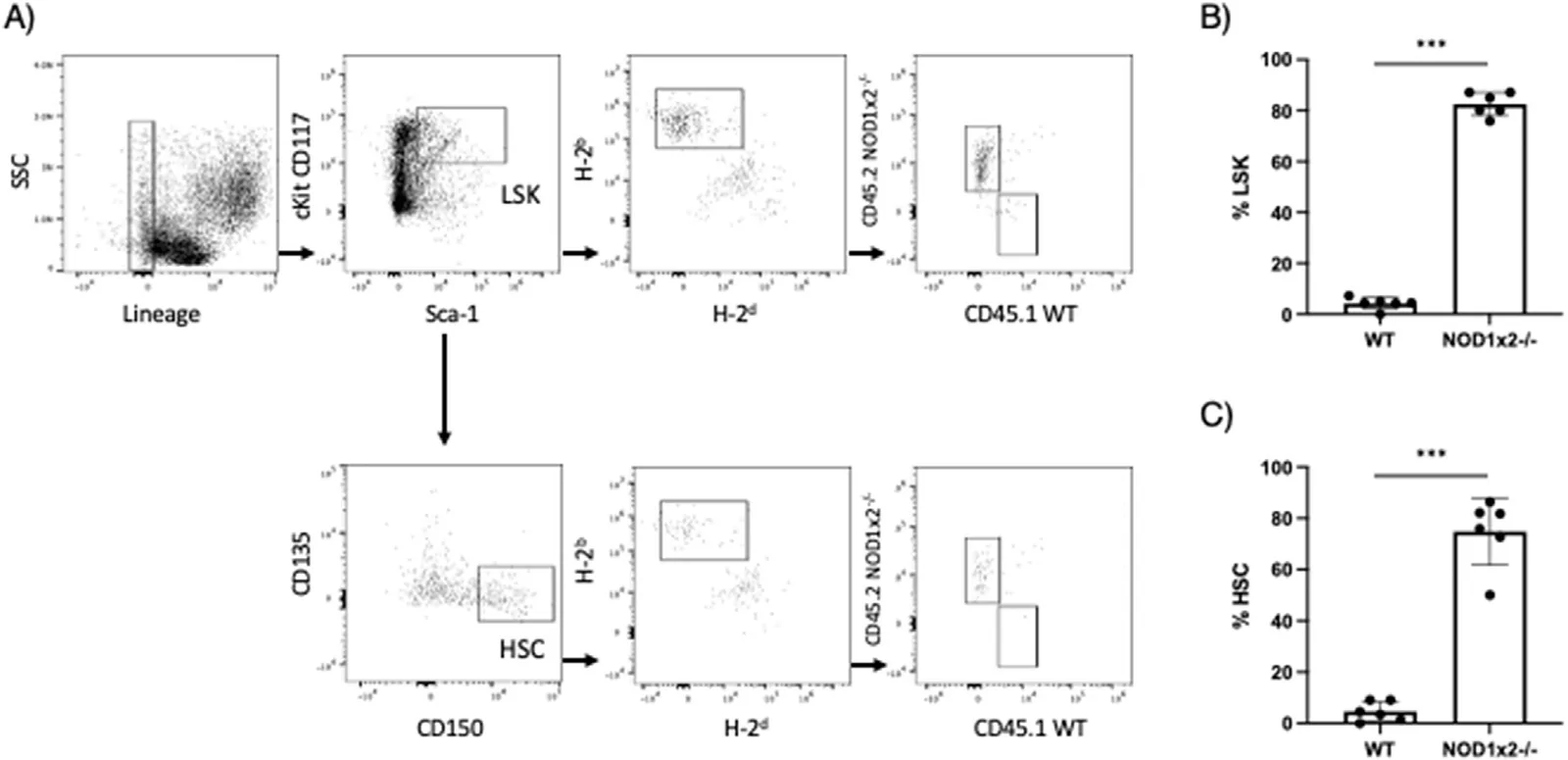
Competitive bone marrow engraftment of WT and NOD1×2^−/−^ donor HSPCs both transplanted into same allogeneic host. (Panel A) Gating scheme for population numbers of WT (B6, CD45.1^+^, H-2K^b^) and NOD1×2^−/−^ (B6, CD41.2^+^, H-2K^b^) donor cells in competitive allogeneic hematopoietic stem cell transplant 6 wk after transplant. (Panel B) Donor LSK+ cells in BALB/c recipient BM 6 wk after transplant. (C) Donor HSPC+ cells in BALB/c recipient BM 6 wk after transplant. n = 9 mice/gp. Error bars are SD, ***p < .001.

**Figure 5.**
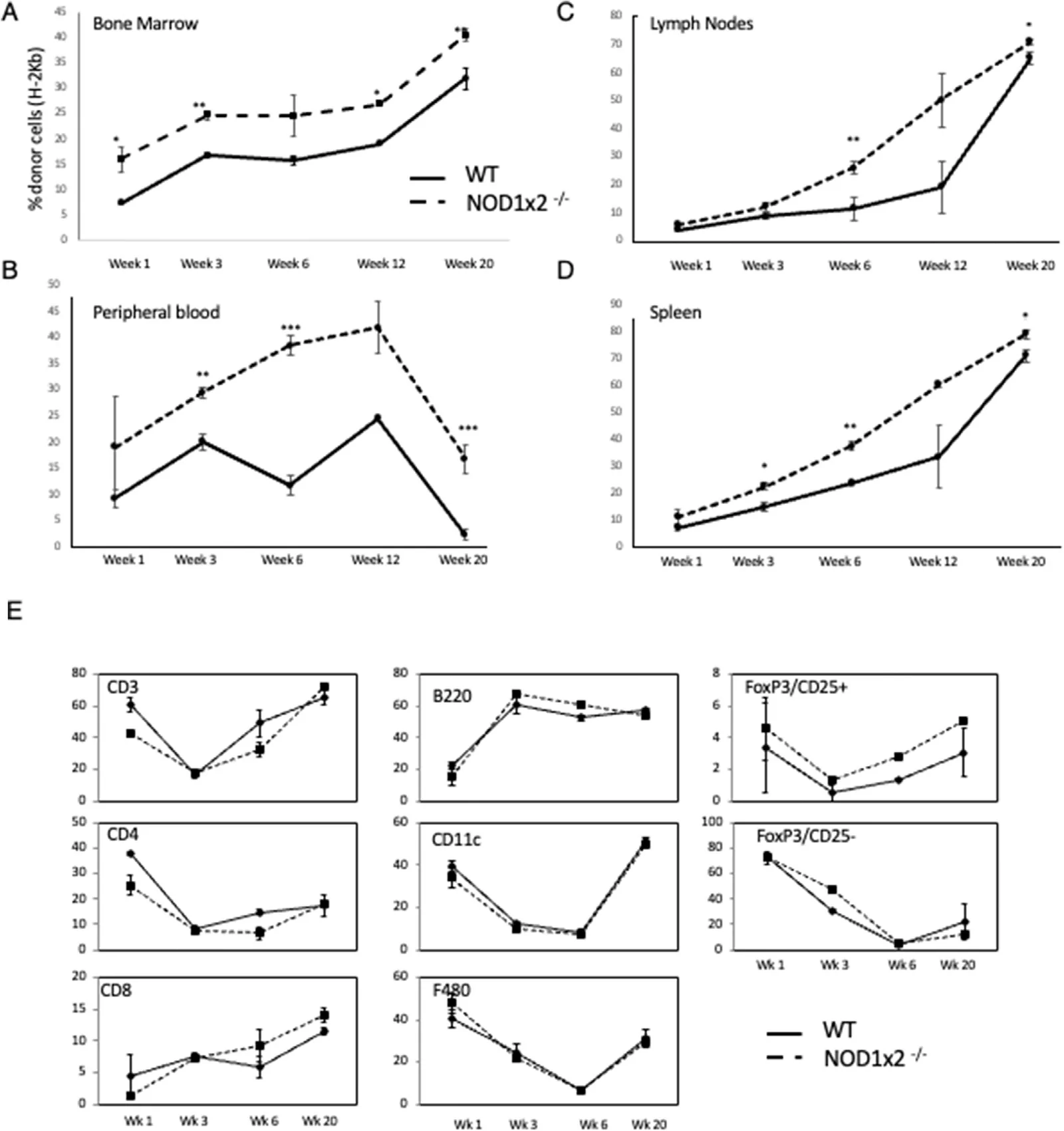
Chimerism in lymphoid tissue of allogeneic recipients transplanted with WT versus NOD1×2^−/−^ donor HSPCs. WT (solid line) or NOD1×2^−/−^ (dashed line) donor cells (both H-2K^b^) detected by congenic antibody staining in bone marrow (Panel A), thymus (Panel B), lymph nodes (Panel C), spleen (Panel D) over time after the allogeneic HSPCs were transplanted into BALB/c (H-2K^d^) recipient mice. (Panel E) shows no significant differences in CD3, CD4, CD8, B220, CD11c, F480, FoxP3/CD25+ or FoxP3/CD25− on peripheral donor cells over 20 wk in the recipients of WT (solid line) versus NOD1×2^−/−^ (dashed line) HSPCs. At each time point 5 WT and 5 NOD1×2^−/−^ chimeric mice were analyzed per group. Error bars are SD, *p < .05, **p < .01.

**Figure 6.**
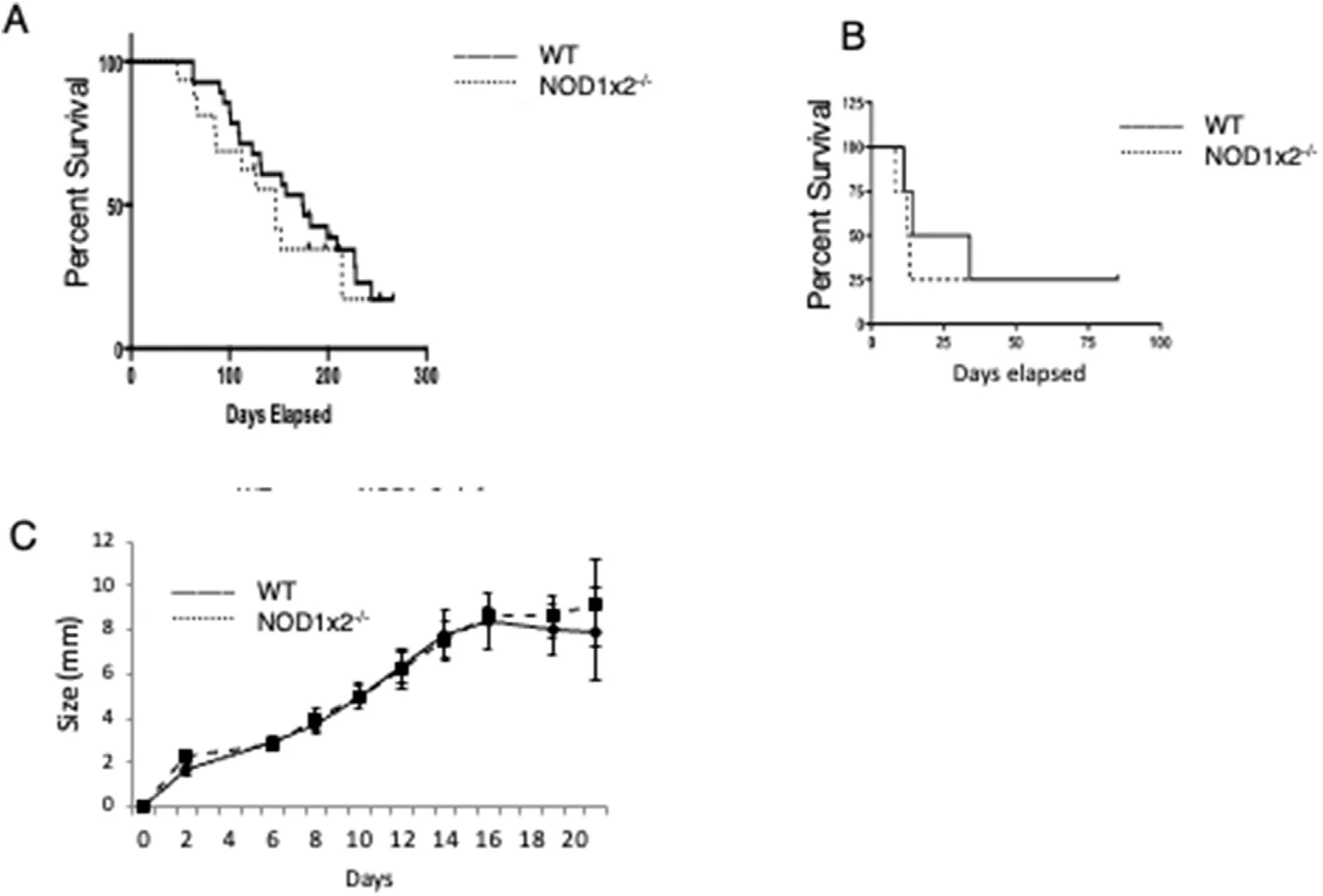
Survival of WT versus NOD1×2^−/−^ chimeric mice and tolerance to skin grafts and to B6F10 (H-2K^b^) tumor cells. (Panel A) Survival of BALB/c recipient (H-2K^d^) mice transplanted with either WT (sold line) or NOD1×2^−/−^ (dashed line) HSPCs (n = 5 mice/gp). (Panel B) Survival of donor skin from WT mice transplanted onto WT chimeric recipient mice versus donor skin from NOD1×2^−/−^ mice transplanted onto NOD1×2^−/−^ chimeric mice (n = 5 mice/gp). (Panel C) Growth of B6F10 tumor cells (H2K^b^) injected to the dermis of WT (solid line) versus NOD1×2^−/−^ (dashed line) chimeric mice (n = 7 mice/gp). Error bar = SD, *p* = ns.

**Figure 7.**
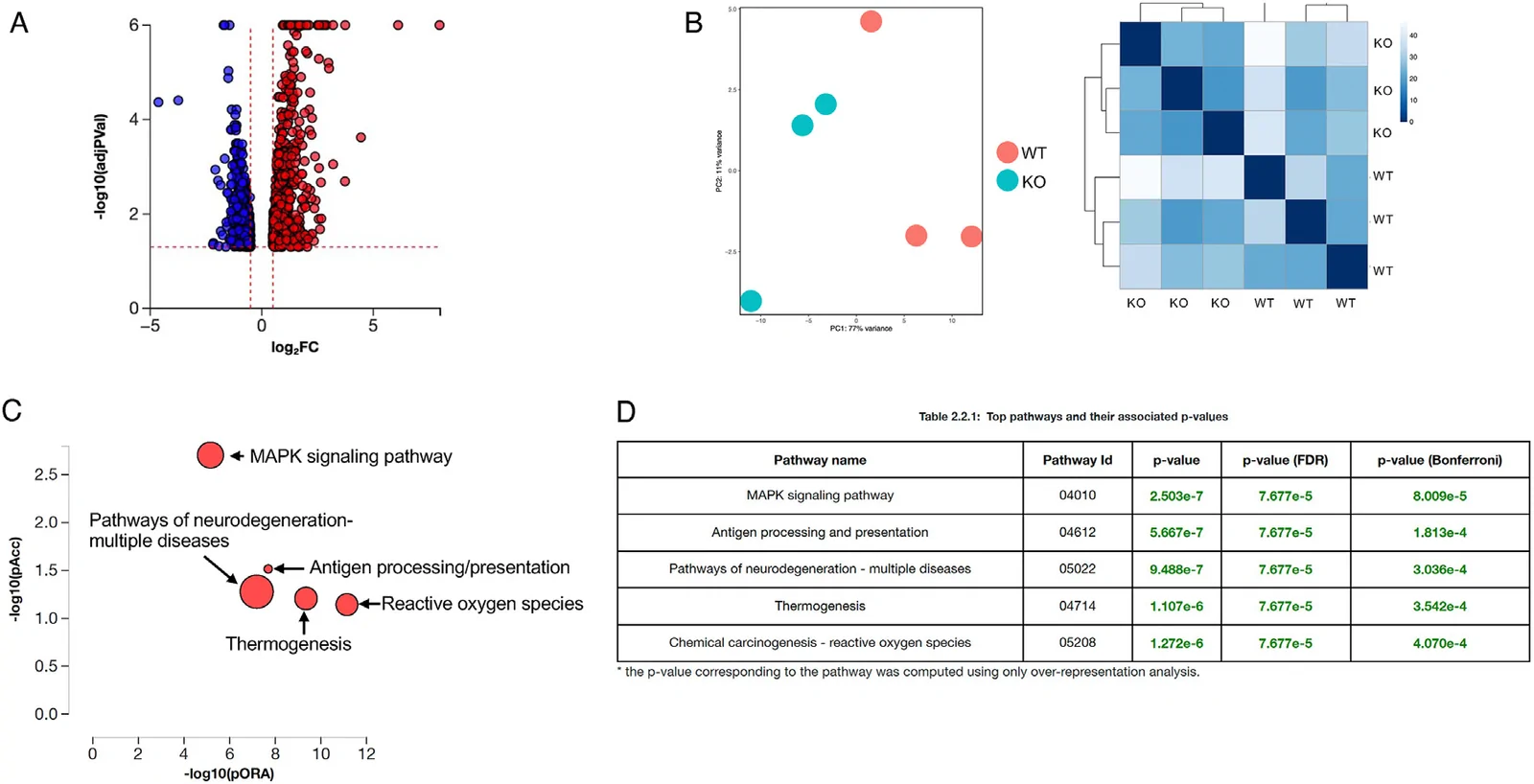

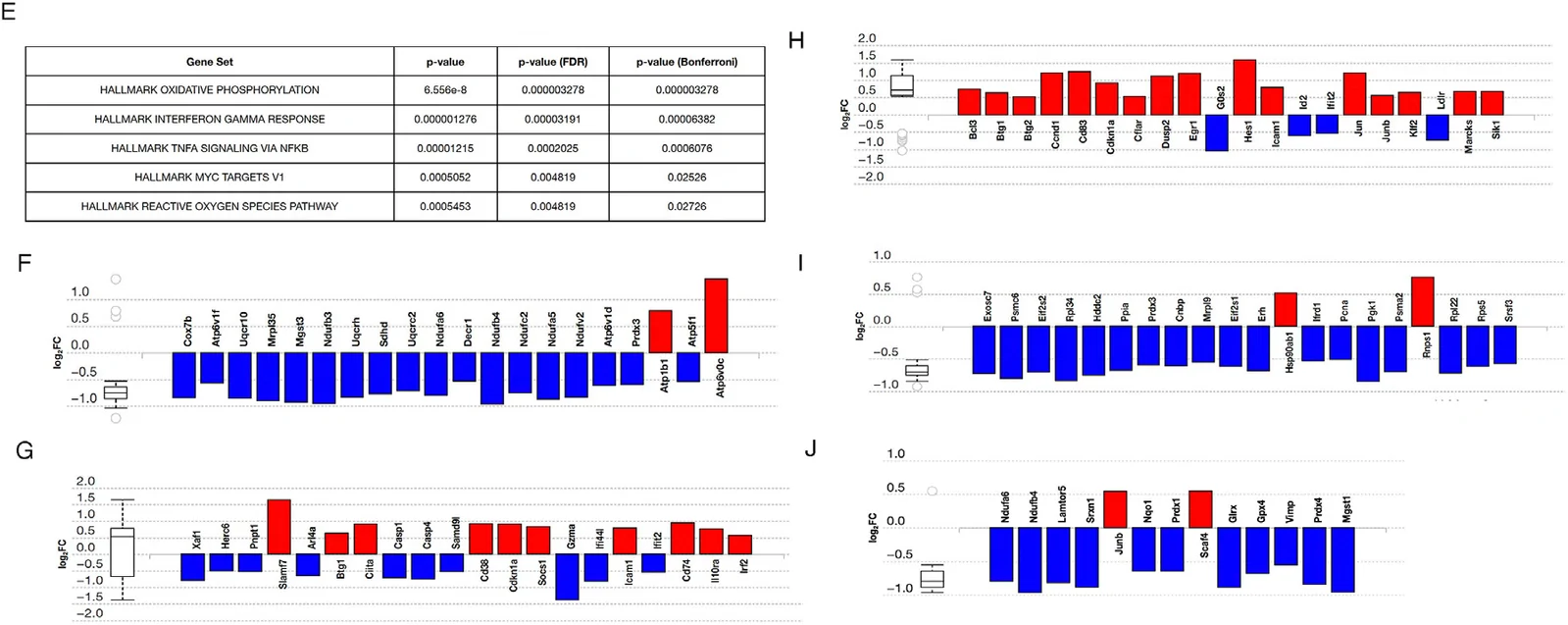
Transcriptomic profiling of donor-derived hematopoietic cells following transplantation. Bulk RNA sequencing was performed on H-2K^b^ donor-derived Lin- cells isolated from BALB/c recipients of WT or NOD1×2^−/−^ HSPCs 6 wk after stem cell transplantation (n = 3 mice/group). (Panel A) Volcano plot of differentially expressed genes comparing WT versus NOD1/2^−/−^donor-derived cells, with upregulated genes shown in red and downregulated in blue. (Panel B, left) Principal component analysis (PCA) demonstrating separation of WT and NOD1/2^−/−^ chimera samples; (right) correlation heatmap confirming within-group clustering. (Panel C-D) Pathway enrichment analysis of differentially expressed genes. Bubble size (C) corresponds to pathway magnitude. The x-axis represents statistical significance (−log_10_ pORA), and the y-axis shows pathway impact (−log_10_ pAcc). The summary table (D) depicts the most significantly enriched pathways among differentially expressed genes based on over-representation and impact analyses. The table *p*-values, false discovery rate (FDR), and Bonferroni-adjusted *p*-values confirm the high statistical significance of these pathways. (Panels E-J) Top enriched Hallmark pathways identified by gene set enrichment analysis (GSEA). *p*-values were adjusted for multiple comparisons using FDR and Bonferroni correction. Top enriched hallmark pathways. include oxidative phosphorylation (F), interferon-*γ* response (G), TNF*α* signaling via NF-*κ*B (H), MYC targets (I), and reactive oxygen species (J) pathways. Corresponding bar plots display log_2_ fold changes of representative genes within each pathway. Blue bars indicate downregulated genes, and red bars indicate upregulated genes. Collectively, these data reveal coordinated alteration in mitochondrial metabolism, inflammatory signaling, and oxidative stress responses in the NOD1×2^−/−^ HSPCs.
