# Supplementary material for: Simultaneous NOD1 and NOD2 Deletion in Hematopoietic Stem and Progenitor cells Promotes Long-Term Donor Chimerism
Source: Transplant Cell Ther. Author manuscript; Available in PMC 2026 Oct 1. (PMC13629068; doi:10.1016/j.jtct.2026.01.031)
Supplement: Supplementary file 1 [file NIHMS2192028-supplement-1.docx]

**Supplemental Figure 1. Lineage cell depletion for WT and NOD1x2^-/-^ donor engrafted cells.** **Panel A.** Gating scheme for pre and post depletion of lineage cells for WT (B6, H-2K^b^) and NOD1x2^-/-^ (B6, H-2K^b^) donor cells prior to allogeneic hematopoietic stem cell transplant. Bone marrow is isolated from WT or NOD1x2^-/-^ mice and negatively depleted of lineage positive cells (CD3e, CD11b, CD19, GR-1, Ter119) with biotinylated antibodies and magnetic particle separation. Pre depleted and post depleted cells were then labeled with a lineage antibody cocktail and cKit (CD117) and analyzed by flow cytometry. Over 90% of cells after depletion were lineage negative cells.
